# Effect of ISBAR Clinical Handover Application on Nurses' Perception of Communication and Attitudes toward Patient Safety at Emirates Maternity Hospital in Gaza Strip, Palestine

**DOI:** 10.4314/ejhs.v33i5.7

**Published:** 2023-09

**Authors:** Yousef Fahajan, Ali Albelbeisi, Yasmin Abu Shnena, Osama J Emad, Deiaa Abu Kweik, Edris Kakemam, Ahmed Hassan Albelbeisi

**Affiliations:** 1 General Directorate of Nursing, Ministry of Health, Gaza, Palestine; 2 Health Research Unit, Ministry of Health, Gaza, Palestine; 3 Faculty of Nursing, Midwifery Department, Islamic University of Gaza, Palestine; 4 General Directorate of Mental Health, Ministry of Health, Gaza, Palestine; 5 Emirate Obstetric Hospital, Ministry of Health, Gaza, Palestine; 6 Clinical Research Development Unit of Tabriz Valiasr Hospital, Tabriz University of Medical Sciences, Tabriz, Iran; 7 Medical Services Directorate, Gaza Strip, Palestine; 8 College of Health Professions, Israa University, Gaza, Palestine

**Keywords:** ISBAR, Clinical handover, Application, Patient Safety

## Abstract

**Background:**

Good communication is necessary for safety and quality of health. This study aims to determine the effect of ISBAR communication on nurses' perception of communication and attitudes toward patient safety in the Emirates Maternity Hospital in the Gaza Strip, Palestine

**Method:**

A single-group hospital-based intervention study (pre and posttest) was conducted. A census sample was used Participants opinions about the effect of ISBAR were gathered using two tools established by Shortell, Rousseau, Sexton, and Helmreich to assess the communication awareness and nurses' attitudes towards safety, respectively, before and after the use of the ISBAR program.

**Results:**

After the ISBAR application, nurses' perception of communication demonstrated a positive and significant increase in the three sub-items (openness, accuracy and understanding, and shift communication) in the nurse-nurse communication. Moreover, in four sub-items (openness, accuracy, and understanding, timeliness, and satisfaction) in nurse–doctor communication, (p < 0.05). Further, the nurses' attitudes toward patient safety showed a significant and positive increase in teamwork climate (p<0.001), safety climate (p = 0.007), job satisfaction and working condition (p<0.001), stress recognition (p= 0.008), and perception of management (p = 0.001).

**Conclusion:**

The results provide significant evidence of the positive effects of the ISBAR program in improving nurses' perceptions of communication and attitudes toward patient safety. It is recommended that healthcare providers use ISBAR communication in their practice. Moreover, periodic training programs are required for effective ISBAR communication among the healthcare team.

## Introduction

Clinical handoff is a means of conveying information, responsibility, and accountability among healthcare team members and is an essential phase of patient care (1). This process is regularly performed in the health field, particularly in the context of shift deliveries, more than twice a day (2). It is a longstanding tradition among healthcare providers and its appropriate and standard practice to deliver safe care (3). It also includes information related to the patient, such as disease diagnosis, hemodynamics, treatment plans, health progress, and changes (4). Accurately sharing information is a key factor in sustaining safe patient care in the healthcare setting, and it is a priority to improve patient safety around the world (5).

Clinical handoff provides advances in nursing services including promoted care, enriched care prioritization, increased transfer of patients' information to nurses, improved documentation, decreased overtime, reduced misinterpretations, and improved efficiency and teamwork (1, 4, 6, 7). Herawati claimed that good handover assist in detecting mistakes and supporting the continuity of care (8).

The shortage of communication between nurses during the handoff process has been recognized as a major cause of poor quality and safety of services and leads to dissatisfaction among clients (9), associated with 80% of major health mistakes and responsible for 20% of adverse complications in patients (10). Imperfect handoffs can increase the risk of adverse complications by not sharing information about vital parts of care, such as early diagnosis, present treatment, and new prescribed drugs (11).

Ineffective handoffs can lead to undesirable problems and threats to patient safety (1). Poor communication during medical handoffs can lead to further complications in the hospital, which is especially problematic in ICUs with high-risk patients (12).

A designed communication tool is useful in sharing patient information effectively, dropping adverse events, supporting patient safety, improving the quality of care, and increasing healthcare providers' satisfaction (13). Moreover, it raises trust among healthcare providers, aids in decreasing errors, and avoids loss of vital information (14). Various measures have been taken in recent decades to improve communication in clinical delivery (10). Identify, Situation, Background, Assessment, and Recommendation (ISBAR) and its derivatives (ISBAR, ISOBAR, and ISOBARR) are one of the standard clinical handoff tools (2) recommended by the World Health Organization (WHO) and the Joint Committee on Health Care for patient clinical handoff (15). These tools contain the patient's current situation (S), the patient's clinical background (B), system status assessment (A), and necessary recommendations (R). To date, no similar study has been conducted in Palestine, and little is known about this issue in Arab countries (16-18). Therefore, the study aims to determine the effect of ISBAR communication on nurses' perception of communication and attitudes toward patient safety in the Emirates Maternity Hospital in the Gaza Strip, Palestine.

## Methods

**Study design**: This single-group hospital-based intervention study (pre and posttest) was conducted from April to July 2022 at Emirates Maternity hospital in Gaza Strip, Palestine among nurses working in all units. ISBAR program was applied only at the Emirates hospital in 2022. A census sample was used to include all nurses and midwives working at the Emirates Maternity hospital.

**Intervention**: The Palestinian Ministry of Health selected this hospital for the study after continuous obstacles in implementing hospital handover policy. All nurses had received an ISBAR training course to enable them to meet these requirements. This policy required the employment of ISBAR to make sure the foremost vital information is handed over during a structured format. Further, at least of one bedside handover in an exceedingly 24-hour period to enable the transfer of patient care needs from one shift to the next and supply a chance for patient and healthcare provider participation in handover. The policy also stipulated that in bedside handover the nurse on the outgoing shift must:

- Introduce the patient and the new shift nurse to each other;
- Focus on communication on the patient's needs;
- Simplify discussion on patient care concerns, state alterations and modifications in offered care;
- Inquire the patient if they have any requests or need clarifications;
- Ask the patient to endorse or clarify information;
- Discuss related charts, treatment plans, or tools in bedside handover.

**Data collection and measures**: Participants' perceptions about the effect of ISBAR were gathered using two tools, i.e. established Shortell, Rousseau (19) and Sexton, Helmreich (20) to assess the communication recognition and nurses' attitudes towards patient safety, respectively, before and after the use of ISBAR technique. The tools were translated into Arabic and then the validity and reliability of these tools were assessed by the test re-test method and Cronbach alpha (0.89, 0.87) methods, respectively.

**Statistical analysis**: Data analysis was performed using SPSS version 24.0. Descriptive statistics (frequency distributions, mean, and standard deviation) were used. ANOVA and Paired t-test were used to determine the effect of ISBAR communication on nurses' perception of communication and attitudes toward patient safety. Fisher's Exact Test was used to determine the differences in the degree of agreement for each item in perception and attitude toward patient safety. A p-value of 0.05 was considered statistically significant.

**Ethics approval**: This protocol of the study is approved by the Ethics committee of Israa University, with the ethics code PHRC/HC/261/22. Further, approval to conduct the study in MOH was gained from the MOH Department of Health Research. All experiments were performed in accordance with the Declaration of Helsinki guidelines and regulations. The researchers explained to the study participants that participation was voluntary and the confidentiality of responses was assured, and a written informed consent form was obtained from subjects.

## Results

Table 1 shows the nurses' perception of communication toward patient safety according to general characteristics. Positive and significant differences were observed in the nurse-nurse perception of communication before and after applying to ISBAR program according to the department and professional position (p<0.001, and p = 0.007, respectively). Moreover, a positive and significant difference (p<0.001) was reported in the nurse-doctor perception of communication before and after the application of ISBAR according to the department only, and nonsignificant differences were observed according to other characteristics such as age, experience, marital status, and educational level. In terms of nurses' attitudes toward patients' safety, positive and significant differences were reported according to department and professional position also (p<0.001, and p = 0.031, respectively), while a nonsignificant effect was observed in other characteristics.

**Table 1:** Nurses' Perception of Communication toward Patient Safety according to General Characteristics (N=51)

Characteristics	N (%)	Nurses' perception about communication								Nurse's attitudes toward Patient Safety			
		Nurse – nurse				Nurse – doctor				Before		After	
		Before		After		Before		After					
		M±SD	F^a^	M±SD	F^a^	M±SD	F^a^	M±SD	F^a^	M±SD	F^a^	M±SD	F^a^
			(p)		(p)		(p)		(p)		(p)		(p)
Department													
Daily Care	05(09.8)	1.50±0.21	0.39	2.83±0.04	9.40	1.44±0.40	0.80	2.96±0.08	5.96	02.0±0.21	1.26	2.53±0.06	5.23
Labor Room	13(25.5)	2.55±0.52	0.876	2.62±0.38	<0.001	2.81±0.32	0.574	2.91±0.19	<0.001	2.38±0.14	0.294	2.41±0.14	<0.001
Supervision Office	09(17.6)	2.75±0.50		2.81±0.14		2.77±0.22		2.85±0.12		2.36±0.24		2.37±0.21	
Obstetrics and Gynaecology	17(33.4)	02.66±01		2.89±0.24		2.81±0.38		2.83±0.35		2.39±0.15		2.49±0.13	
Perioperative department	07(13.7)	1.88±0.67		2.66±0.48		2.05±0.81		2.89±0.26		2.01±0.33		2.52±0.24	
Age													
21 – 30	14(27.5)	2.39±0.62	2.49	2.71±0.46	1.03	2.52±0.76	1.25	2.97±0.08	1.14	2.27±0.31	1.97	2.46±0.16	0.40
31 – 40	26(51)	2.22±0.64	0.071	2.76±0.42	0.385	2.30±0.67	0.300	2.80±0.30	0.341	2.19±0.29	0.131	2.45±0.17	0.750
41 – 50	6(11.8)	2.71±0.39		2.16±1.16		2.78±0.40		2.78±0.40		2.33±0.26		2.37±0.18	
> 50	05(9.8)	2.40±0.82		3±00		2.16±0.83		2.80±0.44		2.24±0.43		2.62±0.20	
Experience													
1 – 5	20(39.2)	2.21±0.64	0.48	2.65±0.48	0.98	2.37±0.73	0.95	2.86±0.30	0.16	2.22±0.31	0.66	2.42±0.17	0.33
6 – 10	7(13.7)	2.25±0.86	0.696	2.71±0.48	0.409	2.37±0.94	0.423	2.97±0.04	0.919	2.21±0.36	0.578	2.52±0.12	0.803
11 – 15	14(27.5)	2.37±0.50		2.85±0.36		2.37±0.54		2.75±0.32		2.21±0.24		2.46±0.17	
> 15	10(19.6)	2.62±0.61		2.60±0.96		2.55±0.69		2.87±0.31		2.32±0.33		2.50±0.23	
Marital Status													
Single	6(11.8)	2.39±0.63	1.61	3±00	0.03	2.53±0.78	0.19	2.91±0.20	0.37	2.31±0.32	0.37	2.47±0.07	0.21
Married	44(86.3)	2.33±0.65	0.211	2.68±0.60	0.964	2.37±0.69	0.826	2.83±0.31	0.688	2.22±0.30	0.381	2.46±0.19	0.811
Divorced	01(02)	02.45		02.00		02.90		02.90		02.31		2.31	
Educational level													
Diploma in Midwifery	2(3.9)	1.95±0.44	0.64	03±0.00	1.22	1.60±0.14	0.40	2.90±0.14	1.61	2.05±0.21	0.14	2.51±0.04	0.93
Bachelor in Nursing	13(25.5)	2.27±0.71	0.634	2.69±0.48	0.313	2.30±0.73	0.804	2.80±0.36	0.187	2.19±0.25	0.965	2.45±0.12	0.452
Bachelor in Midwifery	25(49)	2.27±0.63		2.68±0.47		2.39±0.68		2.83±0.30		2.22±0.33		2.46±0.22	
Bachelor in Nursing and Diploma in Midwifery	06(11.8)	2.83±0.32		2.50±1.22		2.90±0.20		2.98±0.04		2.42±0.21		2.44±0.17	
Master or more	05(9.8)	2.43±0.70		3±00		2.50±0.86		2.86±0.31		2.31±0.36		2.51±0.07	
Professional Position													
Staff	41(80.4)	2.24±0.63	0.20	2.71±0.45	5.49	2.33±0.71	0.14	2.84±0.29	2.46	2.19±0.30	0.16	2.45±0.18	3.74
Head Nurse	01(02)	01.72	0.814	03.0	0.007	01.80	0.868	3	0.096	2.13	0.846	02.55	0.031
Supervisor	09(17.6)	2.62±1.06		2.95±0.08		2.86±0.35		2.86±0.35		2.49±0.15		2.58±0.14	

a One-way ANOVA

As shown in Table 2, after the ISBAR application, nurses' perception of communication demonstrated a positive and significant increase in the three sub-items (openness, accuracy and understanding, and shift communication) in the nurse-nurse communication perception and the four sub-items (openness, accuracy and understanding, timeliness, and satisfaction) in nurse-doctor communication perception also, (p<0.05). Further, the nurses' attitudes toward patient safety showed a significant and positive increase in teamwork climate (p<0.001), safety climate (p = 0.007), job satisfaction and working condition (p<0.001), stress recognition (p = 0.008), and perception of management (p = 0.001).

**Table 2:** Nurses' Perception of Communication and Attitudes toward Patient Safety before and after using ISBAR (N=51)

Variable	Before	After	t	*p* ^a^

	M±SD	M±SD		
**Nurses' perception about communication**				
Nurse – Nurse openness	2.34±0.81	2.96±0.12	5.36	0.001
Accuracy and understanding	2.24±0.60	2.56±0.36	3.15	0.003
Shift communication	2.45±0.70	2.94±0.19	5.13	0.001
Nurse – doctor openness	2.36±0.73	2.90±0.21	5.09	0.001
Accuracy and understanding	2.41±0.66	2.85±0.29	4.45	<0.001
Timeliness	2.35±0.78	2.83±0.35	4.07	<0.001
Satisfaction	2.45±0.71	2.87±0.31	4.08	<0.001
**Nurses' attitudes toward patient safety**				
Teamwork climate	2.13±0.53	2.53±0.28	4.43	0.001
Safety climate	2.34±0.71	2.66±0.24	2.80	0.007
Job satisfaction	2.52±0.56	2.87±0.30	4.56	<0.001
Stress recognition	1.76±0.71	2.52±0.56	-2.76	0.008
Perception of management	2.15±0.49	2.35±0.40	3.47	0.001
Working condition	2.53±0.47	2.75±0.36	3.75	0.001

a Paired t-test

Table 3 shows the nurses' perception of nurse–nurse or nurse–doctor communication before and after using ISBAR. A significant (p = 0.035) and positive increase (37.3%) in the percentage of agreed answers was observed among nurse-nurse when the nurses were asked about the ease of seeking advice from the nurses in this unit. The percentage of agreed answers about those nurses feeling that the nurses of this unit fully understand the information they receive also increased significantly, to 5.9%. Moreover, a significant (p = 0.001) and positive (37.3%, and 31.4%, respectively) increase in the percentage of agreement among the nurse-doctor was observed when the questions were about whether it was easy to talk openly with doctors and the level of communication openness between the nurses and doctors in this unit.

**Table 3:** Nurses' Perception about Nurse-Nurse or Nurse-Doctor Communication before and after using ISBAR (N=**51**)

Communication items	Before				After		*X^2^*	*P^a^*

	Disagree N (%)	Neutral N (%)	Agree N (%)	Disagree N (%)	Neutral N (%)	Agree N (%)		
**Openness (Nurse-Nurse)**								
It is easy for me to talk openly with the nurses in this unit.	13(25.6)	9(17.6)	29(56.9)	00(00)	02(3.9)	49(96.1)	04.27	0.089
Communication between nurses in this unit is very open.	12(23.5)	9(17.6)	30(58.8)	00(00)	02(3.9)	49(96.1)	03.66	0.165
It is easy to ask advice from nurses in this unit.	11(21.6)	10(19.6)	30(58.8)	00(00)	02(3.9)	49(96.1)	05.40	0.035

**Accuracy and understanding (Nurse-Nurse)**								

It is often necessary for me to go back and check the accuracy of information I have received from nurses in this unit.	08(15.7)	07(13.7)	36(70.6)	01(02)	00(00)	50(98)	01.24	01.00
The accuracy of information passed among nurses of this unit leaves much to be desired.	07(13.7)	11(21.6)	33(64.7)	01(02)	00(00)	50(98)	01.19	0.757
I feel that certain this unit nurses completely understand the information they receive.	31(60.8)	05(9.8)	15(29.4)	30(58.8)	03(5.9)	18(35.3)	16.05	0.001
**Shift Communication (Nurse-Nurse)**								
There is effective communication between nurses across shifts.	06(11.8)	09(17.6)	36(70.6)	01(02)	00(00)	50(98)	04.86	0.118
Nurses associated with the unit are well informed regarding events occurring on other shifts.	12(23.5)	11(21.6)	28(54.9)	00(00)	04(7.8)	47(92.2)	10.55	0.001
**Openness (Nurse-Doctor)**								
It is easy for me to talk openly with the doctors in this unit.	12(23.5)	11(21.6)	28(54.9)	00(00)	04(7.8)	47(92.2)	10.55	0.001
Communication between nurses and doctors in this unit is very open.	10(19.6)	13(25.5)	28(54.9)	01(02)	04(7.8)	46(90.2)	12.29	0.001
It is easy to ask advice from doctors in this unit.	08(15.7)	13(25.5)	30(58.8)	00(00)	04(7.8)	47(92.2)	4.20	0.065
**Accuracy and understanding (Nurse-Doctor)**								
It is often necessary for me to go back and check the accuracy of information I have received from doctors in this unit.	10(19.6)	9(17.6)	32(62.7)	01(02)	04(7.8)	46(90.2)	16.02	<0.001
Nurses have a good understanding of doctors' treatment plans.	05(9.8)	12(23.5)	34(66.7)	01(02)	07(13.7)	43(84.3)	17.85	<0.001
Doctors have a good understanding of nursing objectives.	08(15.7)	10(19.6)	33(64.7)	00(00)	05(9.8)	46(90.2)	15.50	<0.001
There is effective communication between nurses and doctors across shifts.	08(15.7)	17(33.3)	26(51)	00(00)	06(11.8)	45(88.2)	11.18	0.001
Doctors associated with the unit are well informed regarding events occurring on other shifts.	11(21.6)	16(31.4)	24(47.1)	00(00)	12(23.5)	39(76.5)	23.79	<0.001
**Timeliness (Nurse and Doctor)**								
I get information on the status of patients when I need it.	12(23.5)	7(13.7)	32(62.7)	01(2)	05(9.8)	45(88.2)	15.46	0.001
In matters pertaining to patient care, nurses call doctors in a timely manner.	11(21.6)	6(11.8)	34(66.7)	00(00)	5(9.8)	46(90.2)	23.14	<0.001
When a patient's status changes, I get relevant information quickly.	13(25.5)	13(25.5)	25(49)	02(3.9)	10(19.6)	39(76.5)	16.91	0.001
**Satisfaction (Nurse-Doctor and Nurse)**								
I am satisfied with the communication between nurses and doctors	09(17.6)	14(27.5)	28(54.9)	00(00)	07(13.7)	44(86.3)	10.81	0.004
I am satisfied with the nurse-to-nurse communications.	07(13.7)	10(19.6)	34(66.7)	00(00)	06(11.8)	45(88.2)	12.31	0.001

a Statistical analysis using Fisher's Exact Test

Moreover, the participants' demonstrated a significant and positive increase in the percentage of agrees answer in the five sub-items of accuracy and understanding, and timelines among nurse-doctor (p < 0.05). Significant (p = 0.004, and 0.001, respectively) and positive increase (31.4%, and 21.5%, respectively) in the percentage of agreement was also noted when participants were asked about satisfaction with the communication between nurses and doctors and satisfaction with the nurse-to-nurse communications.

Table 4 demonstrates the nurses' attitudes toward patient safety before and after using ISBAR. Significant and positive increases in the levels of agreement were observed in all items of the questionnaire including teamwork climate, safety climate, job satisfaction, stress recognition, perception of management, and working conditions (p<0.05). Although the percentage of agreeing in the participants' answers when asked if they like their job or not was positively increased (27.5%); this increment was not significant (0.533). The highest level of increment in agree percentage in the teamwork and safety climates item was noted when participants asked if it is easy for personnel in this unit to ask questions when there is something that they do not understand (31.4%) and receive appropriate feedback about their performance (35.3%). With regard to the management's perception, the answers to the questions about whether the management is doing a good job or not, and whether or not participants were provided with sufficient and timely information about events in the hospital that might affect my work showed the highest approval rate (21.6%). Asking about all the necessary information for diagnostic and therapeutic decisions is routinely available to them; their agree answer was increased by 25.5%.

**Table 4:** Nurses' attitudes toward patient safety before and after using ISBAR (N=51)

Safety attitudes items	Before			After			*X^2^*	*p^a^*

	Disagree N (%)	Neutral N (%)	Agree N (%)	Disagree N (%)	Neutral N (%)	Agree N (%)		
**Teamwork climate**								
In this unit, it is not difficult to speak up if I perceive a problem with patient care.	31(60.8)	16(31.4)	04(7.8)	33(64.7)	10(19.6)	8(15.7)	28.86	<0.001
Disagreements in this unit are resolved appropriately (i.e., not who is right, but what is best for the patient).	08(15.7)	10(19.6)	33(64.7)	00(00)	06(11.8)	45(88.2)	12.26	0.002
It is easy for personnel in this unit to ask questions when there is something that they do not understand.	16(31.4)	10(19.6)	25(49)	07(13.7)	03(5.9)	41(80.4)	21.85	<0.001
The doctors and nurses here work together as a well-coordinated team.	11(21.6)	12(23.5)	28(54.9)	00(00)	08(15.7)	43(84.3)	12.62	0.001
I have the support I need from other personnel to care for patients.	14(27.5)	13(25.5)	24(47.1)	00(00)	12(23.5)	39(76.5)	11.81	0.002
**Safety climate**								
Medical errors are handled appropriately in this unit.	08(15.7)	13(25.5)	30(58.8)	00(00)	06(11.8)	45(88.2)	15.25	<0.001
In this unit, it is not difficult to discuss errors.	28(54.9)	14(27.5)	9(17.6)	27(52.9)	11(21.6)	13(25.5)	31.22	<0.001
The culture in this unit makes it easy to learn from the errors of others.	09(17.6)	09(17.6)	33(64.7)	01(02)	05(9.8)	45(88.2)	7.30	0.046
I would feel safe being treated here as a patient.	08(15.7)	08(15.7)	35(68.6)	01(02)	06(11.8)	44(86.3)	27.72	<0.001
I receive appropriate feedback about my performance.	15(29.4)	11(21.6)	25(49.0)	03(5.9)	05(9.8)	43(84.3)	15.09	<0.001
I am encouraged by my colleagues to report any patient safety concerns I may have.	9(17.6)	08(15.7)	34(66.7)	02(3.9)	03(5.9)	46(90.2)	08.93	0.035

**Job satisfaction**								

Working in this hospital is like being part of a large family.	08(15.7)	07(13.7)	36(70.6)	02(3.9)	03(5.9)	46(90.2)	15.25	0.001
This hospital is a good place to work.	14(27.5)	11(21.6)	26(51)	02(3.9)	06(11.8)	43(84.3)	15.96	<0.001
Moral in this unit area is high.	03(5.9)	09(17.6)	39(76.5)	02(3.9)	02(3.9)	47(92.2)	14.89	0.002
I like my job.	06(11.8)	10(19.6)	35(68.6)	00(00)	02(3.9)	49(96.1)	1.74	0.533
I am proud to work at this hospital.	04(7.8)	15(29.4)	32(62.7)	02(3.9)	03(5.9)	46(90.2)	15.86	<0.001
**Stress Recognition**								
When my workload becomes excessive, my performance is impaired.	32(62.7)	11(21.6)	08(15.7)	22(43.1)	15(29.4)	14(27.5)	28.73	<0.001
I am less effective at work when fatigued.	28(54.9)	10(19.6)	13(25.5)	22(43.1)	14(27.5)	15(29.4)	33.91	<0.001
I am more likely to make errors in tense or hostile situations.	32(62.7)	13(25.5)	06(11.8)	26(51)	16(31.4)	09(17.6)	28.27	<0.001
Fatigue impairs my performance during emergency situations (e.g., emergency resuscitation, seizure).	37(72.5)	08(15.7)	06(11.8)	27(52.9)	12(23.5)	12(23.5)	27.44	<0.001
**Perception of Management**								
Hospital administration supports my daily efforts.	09(17.6)	19(37.3)	23(45.1)	05(9.8)	13(25.5)	33(64.7)	42.14	<0.001
Hospital management does not knowingly compromise the safety of patients.	23(45.1)	17(33.3)	11(21.6)	25(49)	11(21.6)	15(29.4)	51.34	<0.001
Management is doing a good job.	03(5.9)	23(45.1)	25(49)	00(00)	15(29.4)	36(70.6)	27.32	<0.001
I am provided with adequate, timely information about events in the hospital that might affect my work.	6(11.8)	17(33.3)	28(54.9)	01(02)	11(21.6)	39(76.5)	22.31	<0.001
This hospital constructively deals with problem doctors and employees.	07(13.7)	26(51)	18(35.3)	02(3.9)	23(45.1)	26(51)	42.90	<0.001
The levels of staffing in this clinical area are sufficient to handle the number of patients.	25(49)	12(23.5)	14(27.5)	26(51)	07(13.7)	18(35.3)	59.29	<0.001
**Working Condition**								
This hospital does a good job of training new personnel.	04(7.8)	17(33.3)	30(58.8)	00(0)	13(25.5)	38(74.5)	29.50	<0.001
Trainees in my discipline are adequately supervised.	00(00)	12(23.5)	39(76.5)	00(00)	12(23.5)	39(76.5)	55.65	<0.001
All the necessary information for diagnostic and therapeutic decisions is routinely available to me.	8(15.7)	18(35.3)	25(49)	00(00)	13(25.5)	38(74.5)	6.03	0.041

a Statistical analysis using Fisher's Exact Test

## Discussion

This study is the first to examine the effect of ISBAR Communication on Nurses' Perceptions of Communication and Attitudes toward Patient Safety at Emirates Maternity Hospital in the Gaza Strip, Palestine. The study showed a positive and significant difference in the nurse-nurse perception of communication before and after applying for the ISBAR program according to departments and professional positions. The current findings are consistent with studies of ISBAR in other settings, where it has been showing the effect of ISBAR training in increasing communication content, improving the structure and consistency of delivered information, and ultimately enabling recipients to feel better prepared with necessary information (21, 22). The present results confirmed that nurses can benefit from the training program provided to them. Moreover, the study reported a positive and significant difference in the nurse-doctor perception of communication before and after the application of ISBAR according to the departments only, and non-significant differences were observed according to other characteristics such as age, experience, marital status, and educational level. Further, in terms of nurses' attitudes toward patients' safety, positive and significant differences were reported according to departments and professional position also, while a non-significant effect was observed in other characteristics such as age, experience, marital status, and educational level. This concludes that the participants' awareness of handover ISBAR increased after the training compared with before the program. This result agreed with other studies by Noh and Lee (2018) (23) and Wang et al (2015) (24), which appear to support the effects of the program proposed in this study.

On the other hand, the results showed that after ISBAR application, nurses' perception of communication demonstrated a positive and significant increase in the three sub-items (openness, accuracy and understanding, and shift communication) in the nurse-nurse communication perception and the four sub-items (openness, accuracy and understanding, timeliness, and satisfaction) in nurse-doctor communication perception also. Furthermore, the nurses' attitudes toward patient safety showed significant and positive increase teamwork climate, safety climate, job satisfaction and working condition, stress recognition, and perception of management. These results agreed with another study by Noh and Lee (2020) (25), which found that awareness of handover Situation, Background, Assessment, Recommendation, communication self-efficacy, and satisfaction with handover education gradually increased after each step of the ISBAR program. Also the current study results in the line of Kim Young and Kim, Sug ( 2018) (23) study that showed that nurses' scores of their attitudes towards patient safety a significant increase in the five out of the six categories; perception of management, working condition, safety climate, teamwork climate, and job satisfaction (P-value <0.05). Moreover, previous studies have shown that training programs, team-based learning and role-plays that are designed to improve nursing students' communication skills have a positive effect on communicative self-efficacy (13, 26, 27).

The current study demonstrated that nurses' Perception of Nurse-Nurse or Nurse-Doctor Communication using ISBAR showed a significant improvement in all categories of communication between nurses and doctors; these results are consistent with the findings of a study conducted by Kim Young and Kim, Sug ( 2018) (23) which showed that after applying ISBAR communication education, nurses perceived significant improvement in three of the five categories of communication between nurses and doctors; satisfaction, accuracy, and understanding( P-value <0.05). This indicates the significant effectiveness of ISBAR training among nurses who arrange communication between health professionals.

Moreover, a literature review on nursing handovers strongly suggested that a structured tool be used because various handover mnemonic systems provide learners with memory structures [1]. Similarly, the handover content organized and learned in our study was also based on the ISBARQ elements to improve nurses' perceptions about communication and attitudes toward patient safety, suggesting the need to introduce a structured system in handover education.

This study also showed that the nurse's attitudes toward patient safety after ISBAR use improved significantly in all categories including teamwork climate, safety climate, job satisfaction, stress recognition, perception of management, and working conditions (p < 0.05) These results are consistent with studies conducted by Kim Young and Kim, Sug (2018) (23), Noh and lee (2020) (25).

Possible limitations of the study are the small sample size is one of the important limitations of the study, and this can be attributed to the fact that the Emirates Maternity Hospital is the first hospital in which all nurses and midwives are trained on ISBAR; Further ISBAR programs are needed to train all nurses and doctors.

In conclusion, good communication is essential for safe patient care and health care quality, The ISBAR technique is a simple way to maintain good communication. A handover program using ISBAR improved nurses' perception and attitudes of communication toward patient safety, Positive and significant differences were observed in the nurse-nurse or nurse-doctor perception about communication, before and after applying of ISBAR program. The results provide significant evidence of the positive effects of the ISBAR program in improving nurses' perceptions of communication and attitudes toward patient safety. It is recommended that healthcare providers use ISBAR communication in their practice. Moreover, periodic training programs are required for effective ISBAR communication among the healthcare team.
